# Structural Insights into the Photoactivatable CO Release from Mn-CO and Re-CO Complexes for CO Delivery

**DOI:** 10.3390/ijms27156704

**Published:** 2026-07-27

**Authors:** Tao Wu, Chaoyang Shi, Chenyang Liu, Chenjie Qin, Jiangshan Wang, Yating Pang, Wenjun Gong, Wenming Wang, Hongfei Wang

**Affiliations:** Key Laboratory of Chemical Biology and Molecular Engineering of Education Ministry, Institute of Molecular Science, Shanxi University, Taiyuan 030006, China; 18335430397@163.com (T.W.); pangyating123@163.com (Y.P.); wangwm@sxu.edu.cn (W.W.)

**Keywords:** metal complexes, crystal structures, DFT calculations, CO release, photodynamic, cytotoxicity

## Abstract

Two tri-carbonyl complexes, [Mn(CO)_3_(5cpa)Br] (**1**) and [Re(CO)_3_(5cqn)(OCH_3_)] (**2**), were synthesized, where 5cpa is 5-Cl-2-picolinic acid and 5cqn is 5-Cl-8-Hydroxyquinoline. Their structures were determined using X-ray diffraction techniques. The electronic absorption and IR spectra of the complexes were experimentally measured and theoretically assigned through density functional theory (DFT) calculations. The photo-induced CO release was verified using time-resolved infrared spectroscopy, and the transfer of CO to hemoglobin (Hb) was monitored by UV-vis spectroscopy. The rate of CO release and transfer from Mn complex **1** is significantly faster than that from Re complex **2**. Complex **2** exhibits higher cytotoxicity against HeLa cells than complex **1**, with IC_50_ values of 40.1 μM and 10.6 μM for **1** and **2**, respectively, which decrease to 16.2 μM and 4.9 μM after photo irradiation. Moreover, **1** exhibited a stronger binding constant (*K_b_*) with human serum albumin (HSA) than **2**, with values of 1.6 × 10^6^ and 7.0 × 10^5^ M^−1^, respectively. The structures of HSA complex adducts revealed that both the resulting [Mn(CO)_3_(5cpa)]^−^ and [Re(CO)_3_(5cqn)]^−^ group coordinate with the N atom of His146, while four additional dissociated Mn-CO groups were observed to bind to HSA for complex **1**. This study provides insights into the stability, possible metabolic pathways, and potential applications of these carbonyl complexes.

## 1. Introduction

Carbon monoxide (CO) is an important signaling molecule that maintains numerous physiological processes. As a neurotransmitter, CO at appropriate levels can reduce the severity of brain injury, relax blood vessels, and decrease inflammation [1,2,3]. In vivo, CO is primarily produced through the enzymatic catabolism of heme by heme oxygenase (HMOX or HO). It is also generated via non-enzymatic pathways in certain contexts, such as through lipid peroxidation, the action of cytochrome P450 reductase, and other oxidative mechanisms [4,5].

CO holds potential in treating bacterial infections, chronic wounds, myocardial infarction, ischemia, and various other diseases. However, its clinical applications as a therapeutic agent are limited due to its gaseous nature, short half-life, and broad physiological roles. In recent years, various types of organic and inorganic CO-releasing molecules (CORMs) have been extensively used in research on the biology of carbon monoxide. One strategy for applying gasotransmitters in biomedicine is to achieve localized delivery via complexation with metal ions. The inorganic CO donors are primarily composed of carbonyl complexes of iron, iridium, ruthenium, cobalt and Group 6 metals [6].

Additionally, triggering CO release from metal-based CORMs with light enables facile spatial and temporal control of CO liberation. These complexes, which are suitable for light-stimulated CO release, are referred to as photoCORMs [7,8,9]. The cold light-activated CORMs [Mn_2_(CO)_10_] (CORM-1) and Fe(CO)_5_ were proposed, and the reactivity of the Ru-based CORM [RuCl(μ-Cl)(CO)_3_]_2_ (CORM-2) was explored. Mechanistic studies indicated that they release CO via ligand exchange. Moreover, the water-soluble [RuCl(glycinato)(CO)_3_] (CORM-3) and [Mn(CO)_4_{S_2_CNMe(CH_2_CO_2_H)}] (CORM-401) were investigated to release CO in biological systems [10,11,12,13,14,15,16]. CORMs showed a variety of therapeutic applications, including anti-inflammatory, antibacterial, angiogenic, anti-carcinogenic, anti-metastatic, anti-ischemic and cardioprotective properties [17,18,19,20,21,22,23,24,25].

In recent years, the photodynamic processes of many tri-carbonyl complexes with different ligands have been explored [26,27,28,29,30,31,32,33]. These studies have demonstrated that the coordination of ligands strongly modulates the photophysical and biological properties of metal-based photoCORMs. Further research investigating the CO-donating properties of these CORMs suggests that greater attention should be paid to both the released CO and the residual fraction of the CORMs, and it is necessary to clarify their potential binding modes with proteins in vivo. Herein, two tri-carbonyl complexes, [Mn(CO)_3_(5cpa)Br] (**1**) and [Re(CO)_3_(5cqn)(OCH_3_)] (**2**), were synthesized to examine relationships between structural features of the CO donor and induced cellular toxicity upon light-triggered CO release. Both 5cqn (5-Cl-8-Hydroxyquinoline) and 5cpa (5-Cl-2-picolinic acid) are small bioactive molecules with antibacterial and antitumor activities [34,35,36,37,38,39,40,41]. They are widely used as N–O atom-containing ligands for metal ion complexation and have promising biomedical applications.

**Scheme 1:**
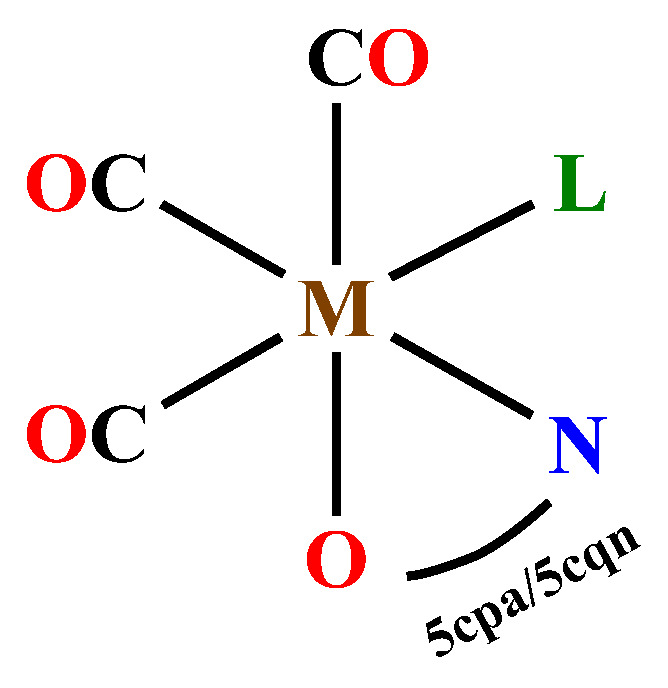
Coordinated structures of complexes **1** and **2** (L is -Br or -OCH_3_; O-N is 5cpa or 5cqn).

The schematic structure of complexes **1** and **2** is shown in Scheme 1. The complexes were characterized by ^1^H-NMR and electrospray ionization mass spectrometry (ESI-MS), and their structures were determined with X-ray crystal diffraction techniques. The UV-vis spectrometry and infrared spectrometry (IR) results of the complexes were analyzed and assigned with density functional theory (DFT) calculations. Moreover, the light-induced CO release from the complexes was examined using time-resolved IR, and the transfer of CO to hemoglobin (Hb) was monitored using UV-vis spectroscopy. The cytotoxicity of the complexes was evaluated against HeLa cells without and upon photo irradiation. Furthermore, the interaction between the complexes and HSA was investigated through a structural viewpoint. This study provides insight into the structure–activity relationship of these complexes, and will promote their potential applications in chemical biology and biomedicine.

## 2. Results

### 2.1. Synthesis and Characteristics of ***1*** and ***2***

Complex **1** was synthesized through the reaction of the 5-chloropyridine-2-carboxylic acid ligand with manganese pentacarbonyl bromide. In contrast, complex **2** was obtained by the reaction of the 5-chloro-8-hydroxyquinoline ligand with rhenium pentacarbonyl chloride. They were separated, purificated and further confirmed by ^1^H NMR and ESI-MS analyses (Figures S1 and S2). The complex dissolved in mixed solvents of methanol or ethanol and dichloromethane at molar ratio of 1:1–1:3, and yellow block-shaped crystals were obtained at room temperature. The crystal structures of the complexes were determined using X-ray diffraction techniques.

**Figure 1:**
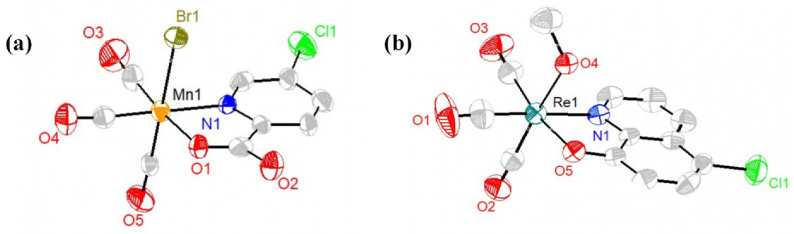
Crystal structures of the two complexes: (**a**) **1**, (**b**) **2**.

The ORTEP diagram illustrating the molecular structures of these complexes is shown in Figure 1, and the detailed crystal structure parameters are listed in Table S1. Complex **1** belongs to the triclinic crystal system with a space group of *C*^−^1. The central metal Mn atom coordinates with three carbonyl groups and one Br atom. Meanwhile, the other two carbonyl groups are respectively replaced by the N and O atoms on the pyridine ring of the 5cpa ligand, forming an octahedral structure. Complex **2** belongs to the monoclinic crystal system with a space group of *I*2/a. The central metal Re atom is hexacoordinated, coordinating with three carbonyl groups and the N and O atoms from the quinoline ring of the 5cqn ligand. Interestingly, one chlorine atom is replaced by a methoxy group derived from the methanol solvent.

### 2.2. Theoretical Calculations

Table S2 lists the calculated bond lengths and angles between the metal and coordinating atoms for the two complexes, and the experimental results obtained through X-ray diffraction. Table S3 lists the experimental maximum absorption wavelengths and major transition bands for the two complexes. In general, the calculated values are basically consistent with the experimental values. The calculated HOMO and LUMO frontier orbitals and relative energy levels are shown in Figure S3, and Tables S3 and S4 list the theoretical and experimental values of the absorption peaks in the UV–visible absorption spectra of the two complexes, along with the primary molecular orbitals and their corresponding contribution values from different molecular groups.

**Figure 2:**
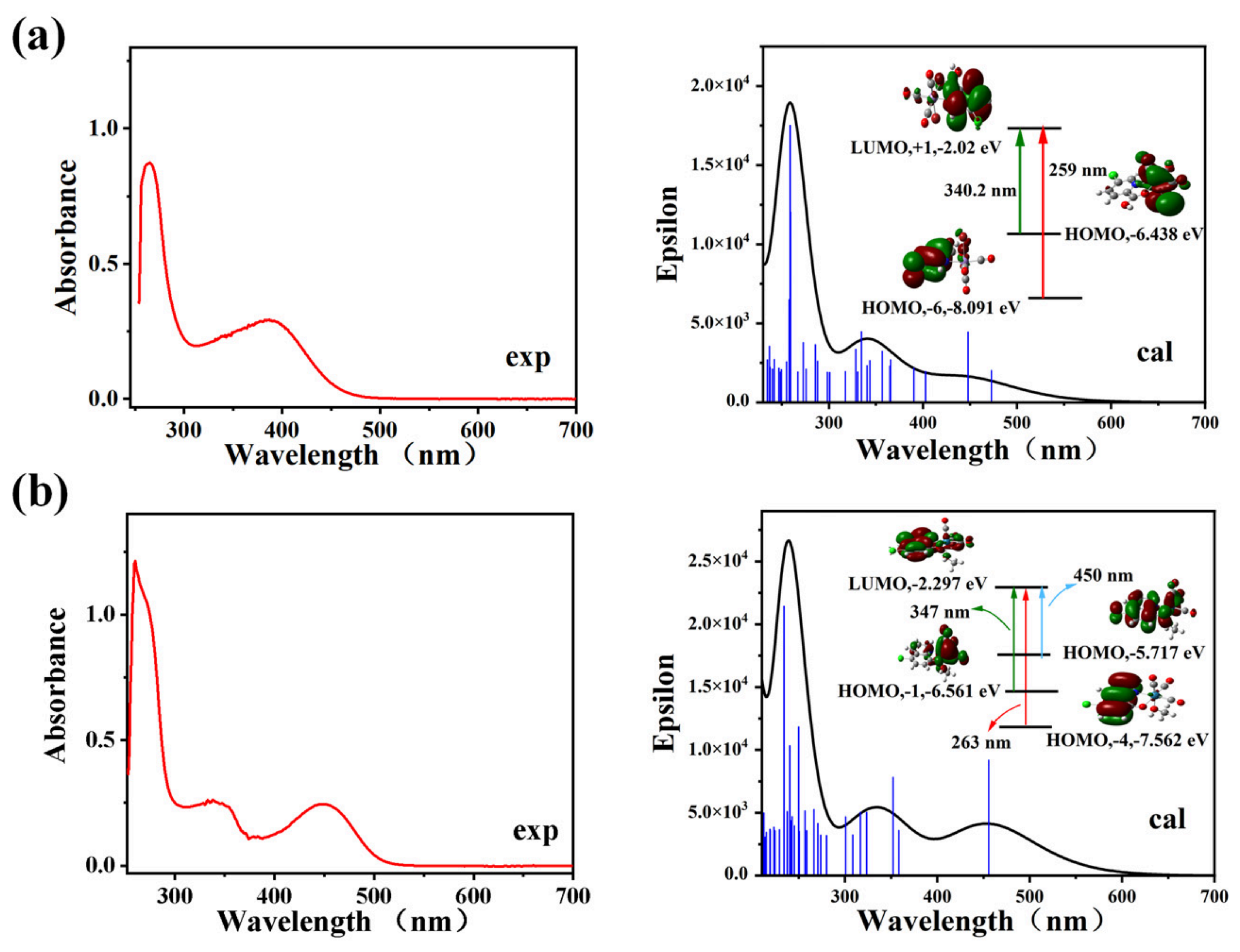
The experimental and calculated UV–visible spectrograms of the complexes: (**a**) **1**, (**b**) **2**.

The calculated and experimental absorption peaks of the two complexes are shown in Figure 2. Complex **1** has two absorption peaks around 265 nm and 392 nm, and the calculated absorption peaks are 258.81 nm and 334.62 nm; they are mainly contributed from orbital transitions by HOMO-6 → LUMO + 1 and HOMO → LUMO + 1, respectively. At 265 nm, both ligand-to-ligand charge transfer (LLCT) and metal-to-metal charge transfer (MMCT) transitions occur. At 392 nm, the transition from the d orbital of Mn (d(Mn)) to the π orbital of 5cpa results in a metal-to-ligand charge transfer (MLCT) transition. Complex **2** shows three absorption peaks around 263 nm, 347 nm, and 450 nm. The calculated absorption peaks are 249.71 nm, 351.97 nm, and 455.56 nm, corresponding to orbital transitions of HOMO-4 → LUMO, HOMO-1 → LUMO, and HOMO → LUMO, respectively. The absorption around 263 nm includes LLCT and LMCT orienting transitions from the π orbital of 5cqn (π(5cqn)) to the d orbital of Re (d(Re)) and π orbital of PA (π(5cqn)). Meanwhile, an MLCT transition occurs around 450 nm and LLCT and LMCT transitions contribute around 347 nm.

**Figure 3:**
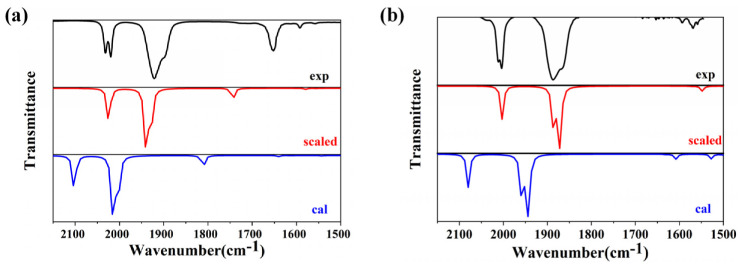
Experimental and calculated values of the infrared spectra for the complexes: (**a**) **1**, (**b**) **2**.

Table S5 lists the experimental wavenumbers of vibration peaks and calculated values of the infrared spectra for the two complexes. As shown in Figure 3, three carbonyl peaks coordinated with a Mn ion appear around 1860 cm^−1^ and 2050 cm^−1^, and one carbonyl peak of 5cpa appears around 1650 cm^−1^; the calculated values match the experimental values. Meanwhile, for the Re complex, two carbonyl vibrational peaks overlapped to form a broad belt from 1830 to 1930 cm^−1^. The third vibration peak of the carbonyl group appears around 2020 cm^−1^. Attributing these peaks is valuable for analyzing the ligand dissociation and the photodynamic release of carbon monoxide. Monitoring the position and intensity of these vibration peaks offers a foundation for studying the mechanisms of complex photoreactions.

### 2.3. Cytotoxicity Assay

Figures S4 and S5 shows the in vitro cytotoxicity and photocytotoxicity of the two complexes evaluated by the CCK-8 method, and the IC_50_ values of the two complexes are listed in Table 1. Different concentrations of complexes were incubated with HeLa cells and HL-7702 cells, and both complexes showed inhibitory effects on cells. In the dark, the IC_50_ values of the two complexes for HeLa cells were 40.11 and 10.57 μM, and they were 42.49 and 15.30 μM for HL-7702 cells. Under illumination, the IC_50_ values of the two complexes for HeLa cells were 16.15 and 4.86 μM, and they were 26.74 and 9.90 μM for HL-7702 cells. For the two types of cells, the cytotoxicity of the two complexes under light irradiation is higher than that in the dark. Both the complexes showed slight photodynamic activity. The Re complex showed a little higher cytotoxicity than that of the Mn complex, possibly due to the properties of the central metal and its ligands.

### 2.4. Photodynamic Studies

**Figure 4:**
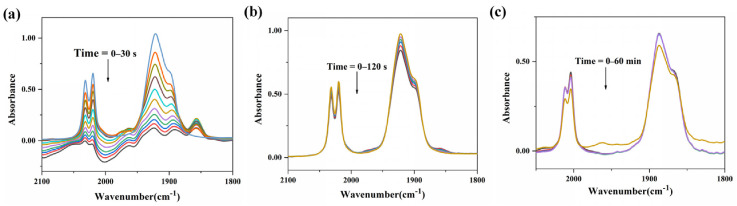
Time-resolution FT-IR spectra of the two complexes in DMSO: (**a**) **1**, λ = 420 nm, (**b**) **1**, λ = 600 nm, (**c**) **2**, white light.

Time-resolved FT-IR spectra. As shown in Figure 4, time-resolved infrared spectroscopy was used to monitored the CO release of the Mn complex upon photo irradiation with 420 nm and 600 nm light. The carbonyl peaks of the complex around 2030 cm^−1^, 2010 cm^−1^, and 1930 cm^−1^ all gradually decreased with the increase in light irradiation time. The rate of CO release for the Mn complex upon 420 nm irradiation is fast and the CO release is complete in approximately 60 s, which is significantly quicker than that upon 600 nm irradiation. Under 600 nm irradiation, CO release proceeds slowly, with only a small change observed after 120 s. Compared with the Mn complex, the Re complex releases CO much more slowly even under stronger white-light irradiation. The decrease in the IR vibrational peak for the Re complex over 60 min is barely close to that of the Mn complex after 2 min. These results indicate that the intensity of light irradiation affects the release rate of CO, and the Mn complex exhibits faster photodynamic properties of CO release than the Re complex, which is consistent with the reported work [29,30,31,32,33].

**Figure 5:**
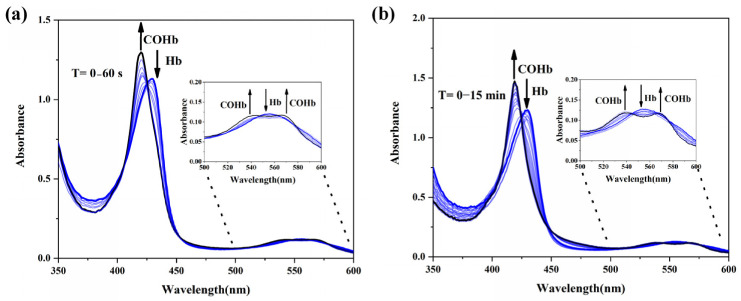
UV–visible spectra monitoring the released CO transfer to hemoglobin of the two complexes upon photo irradiation: (**a**) **1**, (**b**) **2**.

Detection of CO release using hemoglobin. Based on the affinity of hemoglobin (Hb) with carbon monoxide (CO), forming carboxyhemoglobin (HbCO), a typical analysis for detection of released CO transfer to hemoglobin was conducted. As shown in Figure 5, the absorbance of hemoglobin (Hb) decreased at 430 nm and 555 nm upon photo irradiation, while it increased at 410 nm, 539 nm, and 572 nm. The *t*_1/2_ for release and transfer of CO for the Mn complex is about 60 s under 420 nm irradiation, while it is 15 min for the Re complex under white-light irradiation. It is consistent with the previous time-resolved infrared detection for the Mn complex.

### 2.5. Binding with HSA

**Figure 6:**
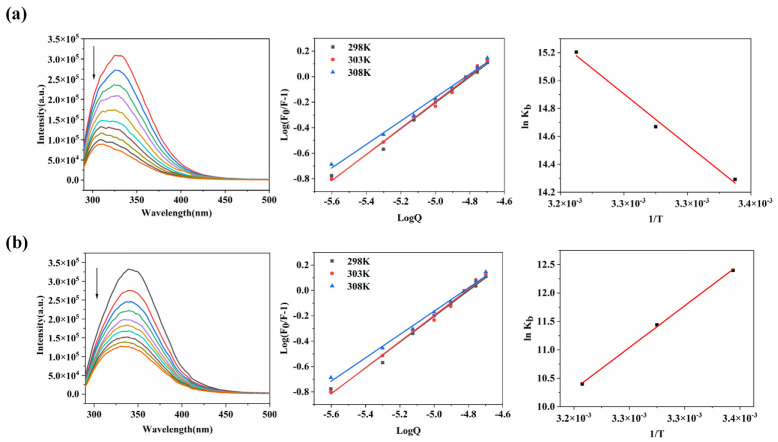
Fluorescence spectra of HSA titrated with different concentrations of complexes **1**–**2** (**left**); Stern–Volmer curves at different temperatures (upper right corner); log [*F*_0_/*F*-1] and log [Q] plots at different temperatures (**middle**); Van’t Hoff plot (**right**): (**a**) **1**, (**b**) **2**.

Fluorescent spectra. HSA can alter the biocompatibility and immune compatibility of drugs, and binding with HSA helps to elucidate their potential metabolic and kinetic processes in vivo. Human serum albumin (HSA) exhibits a characteristic fluorescence spectrum around 345 nm when excited at 280 nm. As shown in Figure 6, with the addition of the complex, the fluorescence emission intensity of HSA at 345 nm decreases significantly. This indicates that an interaction occurs between the complex and HSA, which changes the microenvironment of chromophore residues.

As the temperature increased, the quenching constant *K_sv_* between the two complexes and HSA decreased, and *K_q_* is much larger than the maximum diffusion collision quenching rate constant (2.0 × 10^10^ M^−1^s^−1^), indicating that static quenching is the main quenching mode. From a thermodynamic perspective, the quenching mechanism is characterized by Δ*H* > 0, Δ*S* > 0 and Δ*G* < 0, which reveals that the interaction between the Mn complex and HSA mainly relies on hydrophobic effects, and the process is an endothermic spontaneous reaction. For the Re complex, the quenching mechanism is characterized by Δ*H* < 0, Δ*S* < 0 and Δ*G* < 0, which indicates that the interaction between the Re complex and HSA mainly relies on hydrogen bonds and van der Waals forces, and the process is an exothermic spontaneous reaction.

**Figure 7:**
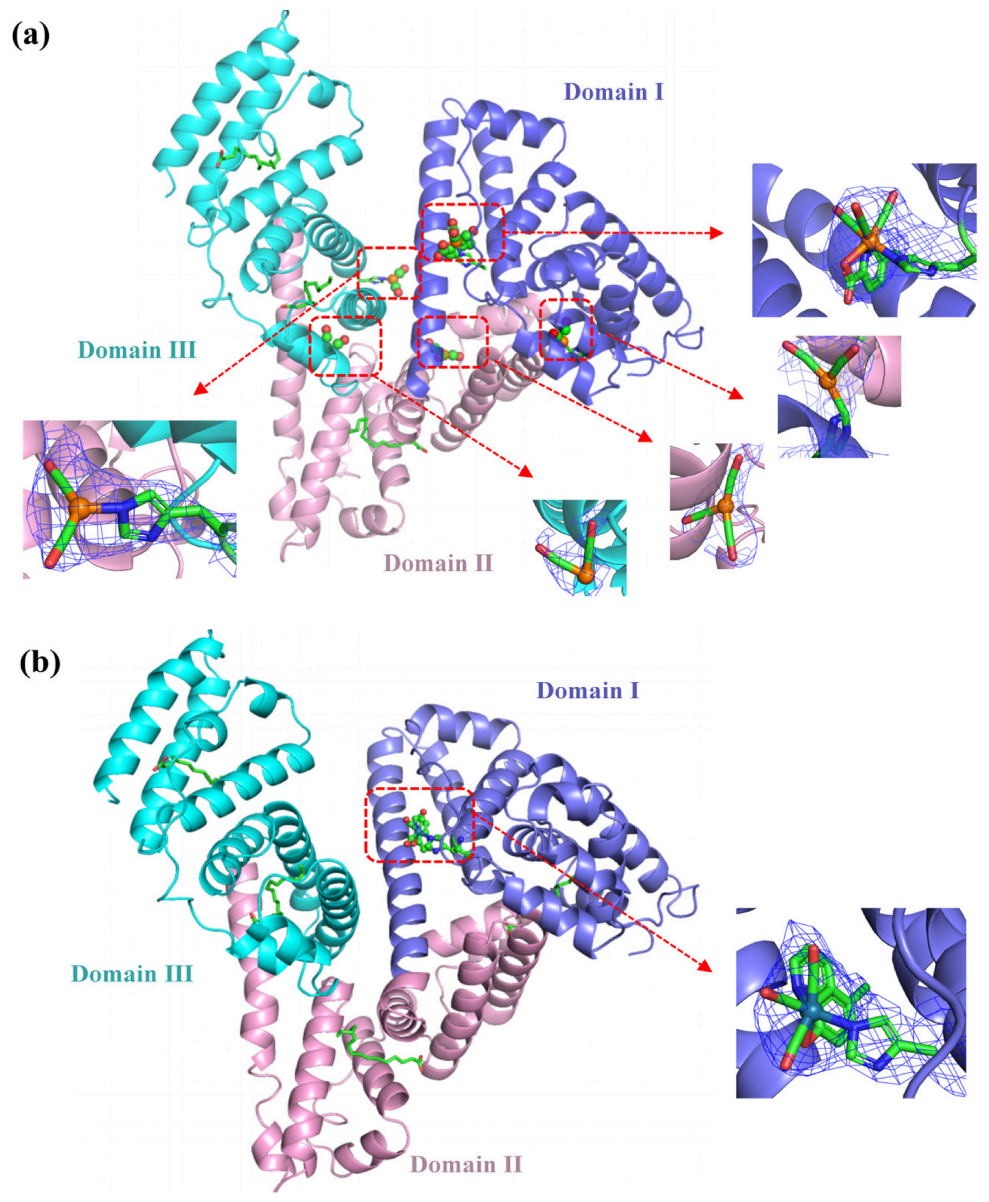
Overall structure of the HSA and complex **1** (**a**) and **2** (**b**) adducts. Every domain is differently colored (domain I, blue; domain II, wheat; and domain III, cyan), and the five palmitic acid (PA) molecules bound to HSA are presented as sticks (aliphatic chain, green; carboxylate oxygen, red). The binding sites of the M-CO complex in HSA are shown with spheres. The density map (2Fo-Fc) for carbonyl complexes is contoured at 1.0 σ and shown as a sky-blue mesh; the ligands are presented as sticks, and Mn and Re atoms are presented in orange and dark blue, respectively.

Structural analysis. Crystal structures of HSA complex adducts were further determined to analyze the interaction model between complexes and HSA and to understand the stability of the complexes. The crystal structure of the HSA complex **1** adduct and complex **2** adduct was solved at a resolution of 2.5 Å and 2.7 Å, respectively. The crystallographic data collection and refinement statistics are presented in Table 2. The overall structure and metal binding site of HSA complex adduct **1** and **2** are depicted in Figure 7.

As illustrated in Figure 7, the crystal structure clearly reveals that one [Mn(CO)_3_(5cpa)] group coordinates with the N atom of His146, and binds to a pocket located in subdomain IB1 of HSA. The surrounding residues of the complex include: Leu154, Lys190, Phe149, Phe157, Arg145, Leu115. Four additional decomposed Mn-CO groups bind in other subdomains between domains I, II and III, and two of them coordinate with His67 and His440, respectively. For the Re complex, one [Re(CO)_3_(5cqn)] group coordinates with the residue His146 and binds to a pocket located in subdomain IB1 of HSA; the surrounding residues of the complex include: Arg186, Lys190, Ser193, Phe157, Leu154, Phe149, Arg145, Tyr161. Clearly, the Mn complex has more binding sites than the Re complex, which is associated with the identity of the central metal and the stability of the complexes.

## 3. Discussion

Two carbonyl complexes [Mn(CO)_3_(5cpa)Br] (**1**) and [Re(CO)_3_(5cqn)(OCH_3_)] (**2**) were synthesized, and their structures were determined with X-ray diffraction techniques. The photo-induced release of CO was proven using time-resolved infrared spectroscopy, and the transferred rate of the released CO to Hb was analyzed semi-quantitatively using UV-vis spectroscopy. The power of the irradiation source obviously influences the rate of CO release, and the excitation wavelength strongly modulates the CO release rate. Under similar conditions, the release and transfer of CO from Mn complex **1** is significantly faster than that from Re complex **2**, which is consistent with previously reported work [33]. Both the Re(I) and Ru(II) analogs exhibited greater stability than Mn(I) photoCORMs. Density functional theory (DFT) calculations provided a better understanding of the electronic absorption and enabled a reasonable assignment for the IR spectra of the complexes to monitor the photodynamic mechanism. Kinetic experiments combined with TD-DFT calculations demonstrated that CO dissociation is triggered by MLCT-induced weakening of M−CO back-bonding, with occasional ligand loss occurring under photo irradiation [29,30,31,32,33].

The cytotoxicity of a series of [Re(CO)_3_(NN)(PR_3_)]^+^ complexes, where NN is a diimine ligand and PR is 1,3,5-triaza-7-phosphaadamantane, tris(hydroxymethyl) -phosphine, or 1,4-diacetyl-1,3,7-triaza-5-phosphabicylco [3.3.1]nonane, was screened. These complexes gave rise to an IC_50_ value of 6 μM in HeLa cells when exposed to irradiation and showed minimal toxicity without light exposure [42]. Both complexes **1** and **2** exhibited photo-enhanced cytotoxicity against HeLa cells, with IC_50_ values ranging from 10 to 40 μM. This value increased to 5–16 μM upon photo irradiation. Furthermore, the structure of HSA complex adducts revealed that complexes **1** and **2** tend to decompose and coordinate with the surface-accessible imidazolyl residues of proteins. This study offers insights into the stability and possible dynamic metabolism of these carbonyl complexes, laying the groundwork for their potential applications.

## 4. Materials and Methods

### 4.1. Materials and Synthesis of Complexes ***1***–***2***

5-Chloropyridine-2-carboxylic acid (5cpa), 5-chloro-8-hydroxyquinoline (5cqn), rhenium pentacarbonyl chloride, manganese pentacarbonyl bromide, and sodium dithionite (SDT) were all procured from Aladdin (Shanghai, China). Hemoglobin (HB), Dulbecco’s Modified Eagle’s Medium (DMEM), fetal bovine serum (FBS), penicillin/streptomycin and PBS buffer solution were purchased from Sangon Biotech (Shanghai, China). Cell Counting Kit-8 (CCK-8) was purchased from Boster Biological Technology Co., Ltd. (Wuhan, China). Human serum albumin (HSA) was sourced from Salarbio (Beijing, China). Human cervical cancer cells (HeLa cells) and normal human liver cells (HL-7702 cells) were procured from the Shanghai Institute for Biological Sciences, Chinese Academy of Sciences (Shanghai, China). All other chemicals and solvents were acquired from local suppliers.

Complex **1**: Mn(CO)_5_Br (2 mmol) and 5-chloropyridine-2-carboxylic acid (2 mmol) were added to a mixed solution of methanol (25 mL) and n-hexane (5 mL). The reaction mixture was refluxed at 65 °C under a nitrogen atmosphere for 2 h. Then, the solvent was removed by rotary evaporation. Complex **2**: Re(CO)_5_Cl (2 mmol) and 5-chloro-8-hydroxyquinoline (2 mmol) were added to methanol (30 mL), and subsequently refluxed at 65 °C for 6 h. The solvent was similarly removed. Both complexes were further purified via recrystallization.

Complex **1**. ^1^H NMR (600 MHz, DMSO-d6): δ 9.01 (d, J = 1.1 Hz), 8.32 (dd, J = 4.1, 1.1 Hz), 7.97 (d, J = 4.1 Hz). ESI-MS (MeOH): calculated m/z for C_9_H_4_BrCINO_5_Mn: 373.8261; found: 373.8287.

Complex **2**. ^1^H NMR (600 MHz, DMSO-d6): δ 9.07 (dd, J = 4.9, 1.3 Hz), 8.67 (dd, J = 8.6, 1.3 Hz), 7.78 (dd, J = 8.6, 4.8 Hz), 7.64 (d, J = 8.4 Hz), 6.89 (d, J = 8.5 Hz), 5.33 (t, J = 5.0 Hz), 3.17 (s). ESI-MS (MeOH): calculated m/z for C_13_H_9_ClNO_5_Re: 479.9646; found: 479.9658.

### 4.2. X-Ray Crystallography

Single-crystal X-ray diffraction data for two complexes were collected at room temperature using a Bruker D8 Venture diffractometer (Bruker Daltonics Bruck, Bremen, Germany) with graphite-monochromated Mo-Kα radiation (λ = 0.71073 Å). The structures were solved using the SHELXTL-97 software package and refined by full-matrix least-squares methods on F^2^ [43]. The non-hydrogen atoms were refined anisotropically. All hydrogen atoms were positioned in calculated positions with fixed isotropic thermal parameters and were included in the final structure factor calculations. Empirical absorption corrections were applied using the SADABS program [44]. X-ray crystallographic data for the complexes have been deposited with the Cambridge Crystallographic Data Centre (CCDC), with the accession numbers 2295345 (**1**) and 2407113 (**2**).

### 4.3. Quantum Chemical Calculations

Quantum chemical calculations for the two complexes were conducted using Gaussian 09 [45], and the molecular structures along with the computational results were visualized with GaussView 5.0 [46]. The atomic coordinates were derived from the crystal structure determined through X-ray diffraction. The structures of the Mn and Re complexes were fully optimized using the Becke three-parameter hybrid functional combined with the Lee–Yang–Parr correlation functional (B3LYP) at the density functional theory (DFT) level [47,48,49]. A mixed basis set was used for the simulation calculations. In the case of complex **1**, the Mn atom was described using the Aug-cc-pVDZ basis set [50], while the ligand atoms were described using the 6-311++G (d, p) basis set [51]. For complex **2**, the Re atom was described using the LanL2DZ basis set [52], and the ligand atoms were also described using the 6-311++G (d, p) basis set. The Polarized Continuum Model (PCM) was used to simulate solvent effects within a DMSO solution system. Electronic absorption spectra for the two complexes were calculated employing Time-Dependent Density Functional Theory (TD-DFT).

### 4.4. UV-Vis and FT-IR Spectroscopy

The UV-Vis absorption spectra of the Mn and Re complexes (0.05 mM) were measured using a Thermo 220 UV-Vis spectrophotometer (Thermo Fisher Scientific, Delaware, DE, USA) with a scanning range of 250–700 nm. The release of CO induced by light was monitored using time-resolved infrared spectroscopy with a Nicolet IS50R FT-IR spectrometer (Thermo Scientific, Delaware, DE, USA) within the scanning range of 2100–1500 cm^−1^. A solution of the complex (10 mM, 100 μL in DMSO) was introduced into an IR liquid cell. The sample solution was subsequently irradiated with a xenon lamp (300 mW/cm^2^, HSX-F300, Beijing NBeT Co., Beijing, China). During the irradiation of the Mn complex, the lamp was fitted with 420 nm and 600 nm filters; for the Re complex.

### 4.5. Light-Triggered CO Transfer to HB Monitored by UV-Vis Spectrometry

Hemoglobin from bovine blood was dissolved in deoxygenated phosphate-buffered saline (DPBS, 10 mM, pH 7.4) to prepare a hemoglobin solution. Sodium dithionite (1.6 mg) was added to the solution under a nitrogen atmosphere to facilitate reduction. Then, 2 mL of the hemoglobin solution was transferred to a cuvette, followed by the addition of 10 μL of either a Mn or Re complex solution (both complexes dissolved in DMSO, 10 mM). The mixture was then covered with a layer of paraffin oil. The samples were irradiated using a Xe lamp. A 420 nm filter was utilized during the irradiation of the Mn complex, whereas the Re complex was exposed to unfiltered white light. UV-Vis spectra ranging from 350 to 600 nm were recorded at specified time intervals using a UV-Vis spectrophotometer [53,54,55,56].

### 4.6. In Vitro Cytotoxicity Assays

The in vitro cytotoxic activities of Mn and Re complexes were evaluated against HeLa tumor cells and HL-7702 non-tumor cells under dark or light conditions using the CCK-8 assay. The cells were cultured in a humidified incubator set at 37 °C with 5% CO_2_, employing DMEM supplemented with 10% (*v*/*v*) FBS and 1% (*v*/*v*) penicillin/streptomycin. Cells were seeded into 96-well plates at a density of 5 × 10^4^ cells per well. After a 24 h incubation period, the cells were treated with varying concentrations of the two complexes. For the light-exposed groups, cells were irradiated with an LED light source (λ = 420 nm, 100 mW/cm^2^) for 20 min; 2 h later, the complexes were added. After a 24 h incubation period with the complexes, the CCK-8 reagent was added to each well, and the cells were further incubated for an additional 2 h. Absorbance was subsequently measured at 450 nm using a SpectraMax iD 5 (Molecular Devices, San Jose, CA, USA) microplate reader. The IC_50_ values were then calculated using SPSS-25 software. Each experiment was conducted in triplicate.

### 4.7. HSA Binding Assay

The interactions of Mn and Re complexes with HSA were investigated using a fluorescence spectrometer (Edinburgh Instruments, FS5, Livingston, UK). Both complexes were prepared as 10 mM solutions in DMSO. HSA was dissolved in phosphate-buffered saline (PBS, 20 mM, pH 7.4) to a final concentration of 10 µM. The complexes were sequentially added to 2 mL of the HSA solution. Fluorescence emission spectra were recorded at three temperatures (298, 303, and 308 K) following excitation at 280 nm. Emission spectra were monitored from 295 to 500 nm with a fixed slit width of 2 nm for both the excitation and emission monochromators. The binding parameters were determined using the Stern–Volmer equation, *F*_0_*/F* = 1 + *K_SV_* [Q] = 1 + *k_q_ τ*_0_ [Q], and its logarithmic form, log [(*F_0_* − *F*)/*F*] = log *K_b_* + n log [Q]. These analyses were utilized to quantify binding affinity and elucidate the mechanism of fluorescence quenching. Thermodynamic parameters were derived using the van’t Hoff equation, ln *K_b_* = −Δ*H*/(RT) + Δ*S*/R, which helped to clarify the nature of the interacting forces between the complex and HSA (*K_SV_*: Stern–Volmer quenching constant; *k_q_*: quenching rate constant; *τ_0_*: average lifetime of the biomacromolecule in the absence of a quencher; [Q]: concentration of the complex; *K_b_*: binding constant; n: the number of binding sites) [57].

### 4.8. Structural Determination of HSA Complex Adduct

The native crystals of HSA were soaked in a 3 μL droplet solution containing 5 mM of either the Mn or Re complex for 5 h. The solution comprised 25% (*w*/*v*) PEG 3350, 5% DMSO and 25 mM potassium phosphate buffer. X-ray diffraction data were collected at the Shanghai Synchrotron Radiation Facility (SSRF) in Shanghai, China. The X-ray diffraction data were processed using HKL-3000 software (HKL Research, Charlottesville, VA, USA) [58]. The coordinate file was obtained through data restoration and molecular replacement using CCP4 (version 7). The final structure of the HSA and Mn or Re complex adducts was rebuilt using COOT (0.8.9) [59] and refined using the Phenix software package version 1.19.2-4518 [60]. Structural figures were generated using PyMOL-3.1.6.1 software [61].

## Figures and Tables

**Table 1:** IC_50_ values of complexes **1** and **2** against HeLa and HL-7702 cells.

	IC_50_ (μM)	1	2
HeLa	Dark	40.11 ± 2.41	10.57 ± 0.21
	Light	16.15 ± 1.76	4.86 ± 0.15
HL-7702	Dark	42.19 ± 2.66	15.30 ± 1.49
	Light	26.74 ± 1.97	9.90 ± 1.08
SI	Dark	1.06	1.45
	Light	1.66	2.04

**Table 2:** Crystallographic data for the HSA complex **1** and **2** adducts.

	1	2
PDB ID	9XRT	9XU4
a (Å)	186.11	183.53
b (Å)	39.01	38.57
c (Å)	96.43	95.66
α, β, γ (°)	90, 104.24, 90	90, 104.98, 90
Space group	C121	C121
Wavelength used (Å)	0.9791	0.9785
Resolution (Å)	45.20–2.50	36.46–2.72
No. of all reflections	277,479	115,659
No. of unique reflections	68,807	17,667
Completeness (%)	99.6	99.0
Average I/σ (I)	7.3 (0.7)	15.66 (1.63)
R_merge_^a^ (%)	0.12	0.10
No. of reflections used (σ(F) > 0)	23,743 (2471)	17,627 (1055)
R_work_^b^ (%)	25.22	25.64
R_free_^b^ (%)	30.19	31.75
r.m.s.d. bond distance (Å)	0.022	0.005
r.m.s.d. bond angle (º)	0.820	0.759
Average B-factor (Å^2^)	61.3	68.5
No. of protein atoms	4665	4666
No. of solvent atoms	16	7
Rotamer outliers (%) Ramachandran plot	0.41	0.41
Favored (%)	94.14	96.72
Allowed (%)	5.69	2.93
Disallowed (%)	0.17	0.34

^a^ *R_merge_* = *Σ_h_Σ_l_* | *I_ih_*−<*I_h_*> |/*Σ_h_Σ_I_*<*I_h_*>, where <*I_h_*> is the mean of the observations *I_ih_* of reflection h. ^b^ *R_work_* = *Σ*(||*F_p_*(*obs*)|−|*F_p_*(*calc*)||)/*Σ*|*F_p_*(*obs*)|; *R_free_* is an *R* factor for a pre-selected subset (5%) of reflections that was not included in refinement.

## Data Availability

The original contributions presented in this study are included in the article. Further inquiries can be directed to the corresponding authors.

## Supplementary Materials

The following supporting information can be downloaded at: https://www.mdpi.com/article/10.3390/ijms27156704/s1.
