# Supplementary material for: Structural Insights into the Photoactivatable CO Release from Mn-CO and Re-CO Complexes for CO Delivery
Source: Int J Mol Sci. 2026 Jul 27;27(15):6704. doi: 10.3390/ijms27156704 (PMC13466244; doi:10.3390/ijms27156704)
Supplement: Supplementary file 1 [file ijms-27-06704-s001.zip › ijms-4428714-supplementary.pdf]

Structural Insights into the Photoactivatable CO Release from Mn-CO and Re-CO Complexes for CO Delivery

Tao Wu [1]†, Chaoyang Shi [1]†, Chenyang Liu [1], Chenjie Qin [1], Jiangshan Wang [1], Yating Pang [1], Wenjun Gong [1], Wenming Wang [1] and Hongfei Wang [1,*]

[1] Key Laboratory of Chemical Biology and Molecular Engineering of Education Ministry, Institute of Molecular Science, Shanxi University, Taiyuan 030006, China
* Correspondence: wanghf@sxu.edu.cn
† These authors contributed equally to this work.

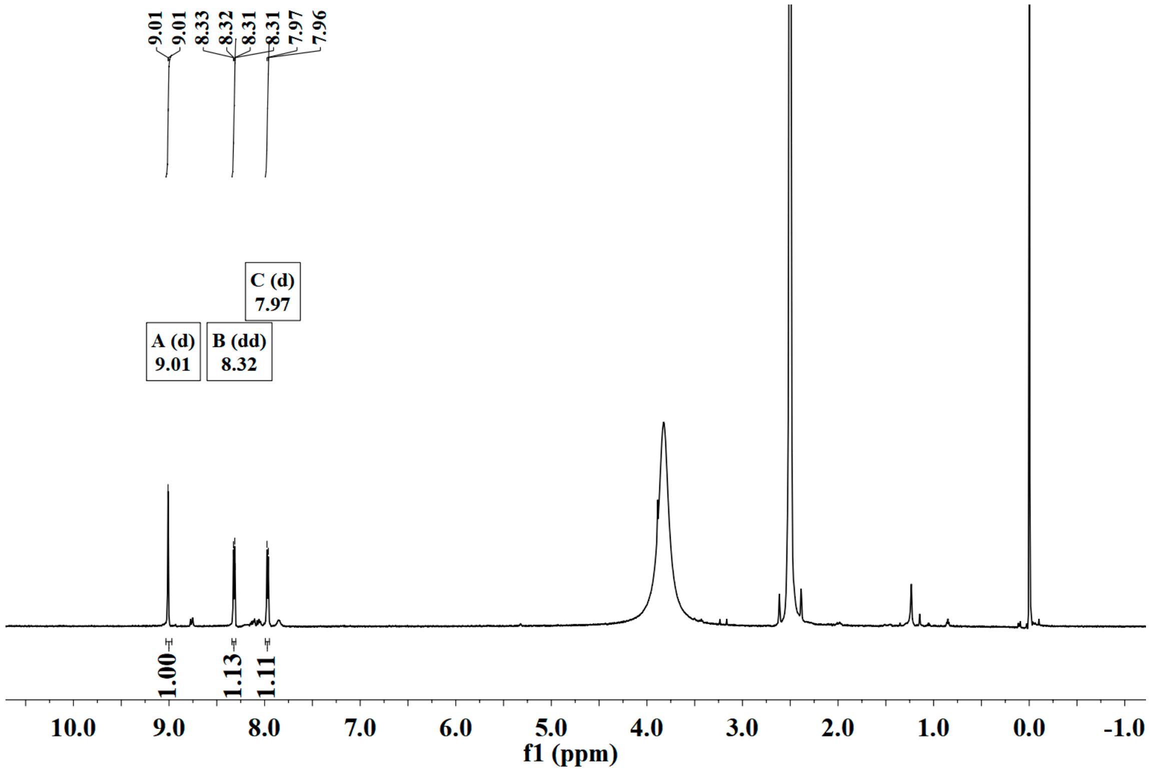

(a)

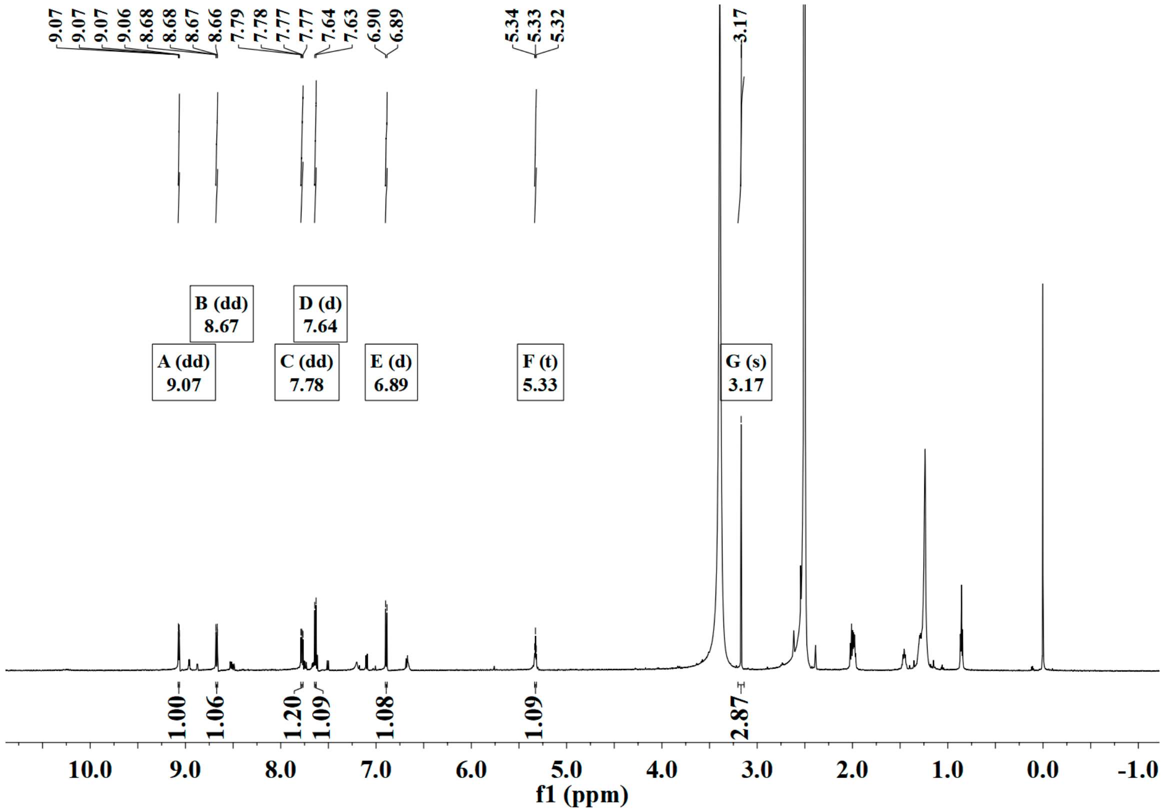

(b)

Figure S1. ^{1}H NMR spectra of the two complexes in DMSO-d_6: (a) complex **1**, and (b) complex **2**.

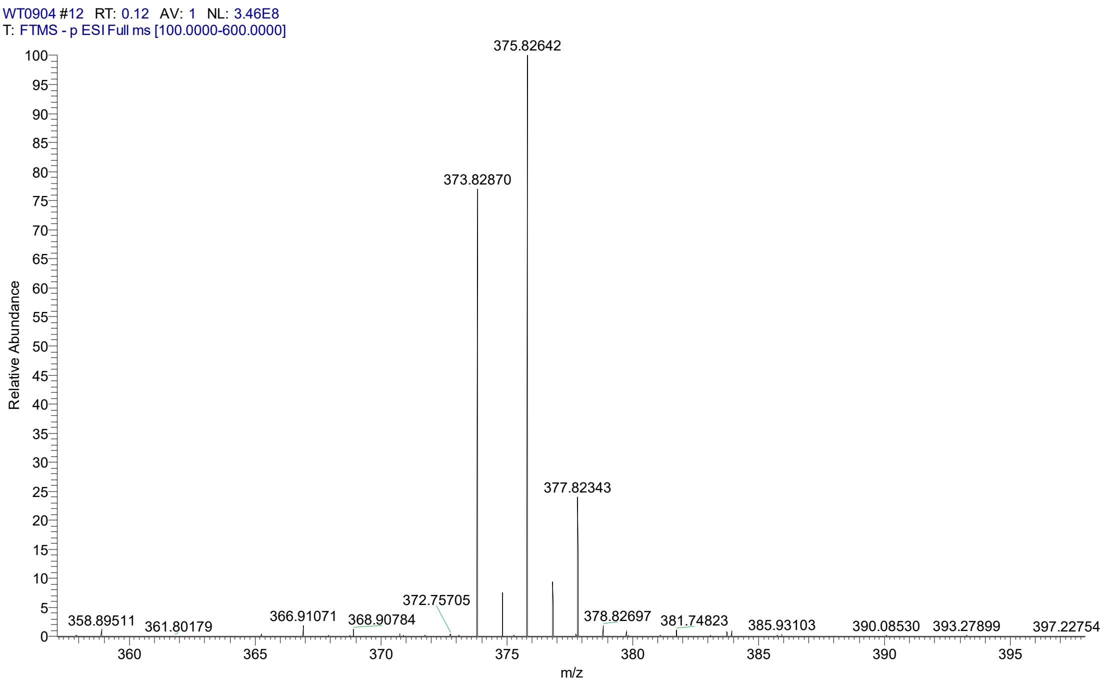

(a)

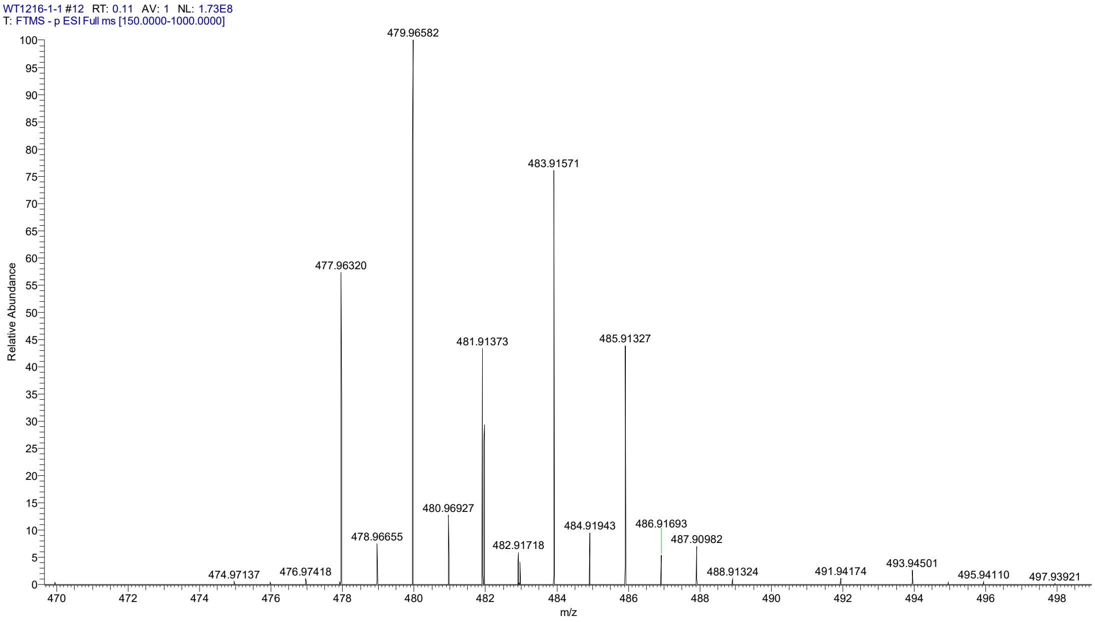

(b)

Figure S2. ESI-MS spectra of the two complexes in methanol: (a) complex **1**, and (b) complex **2**.

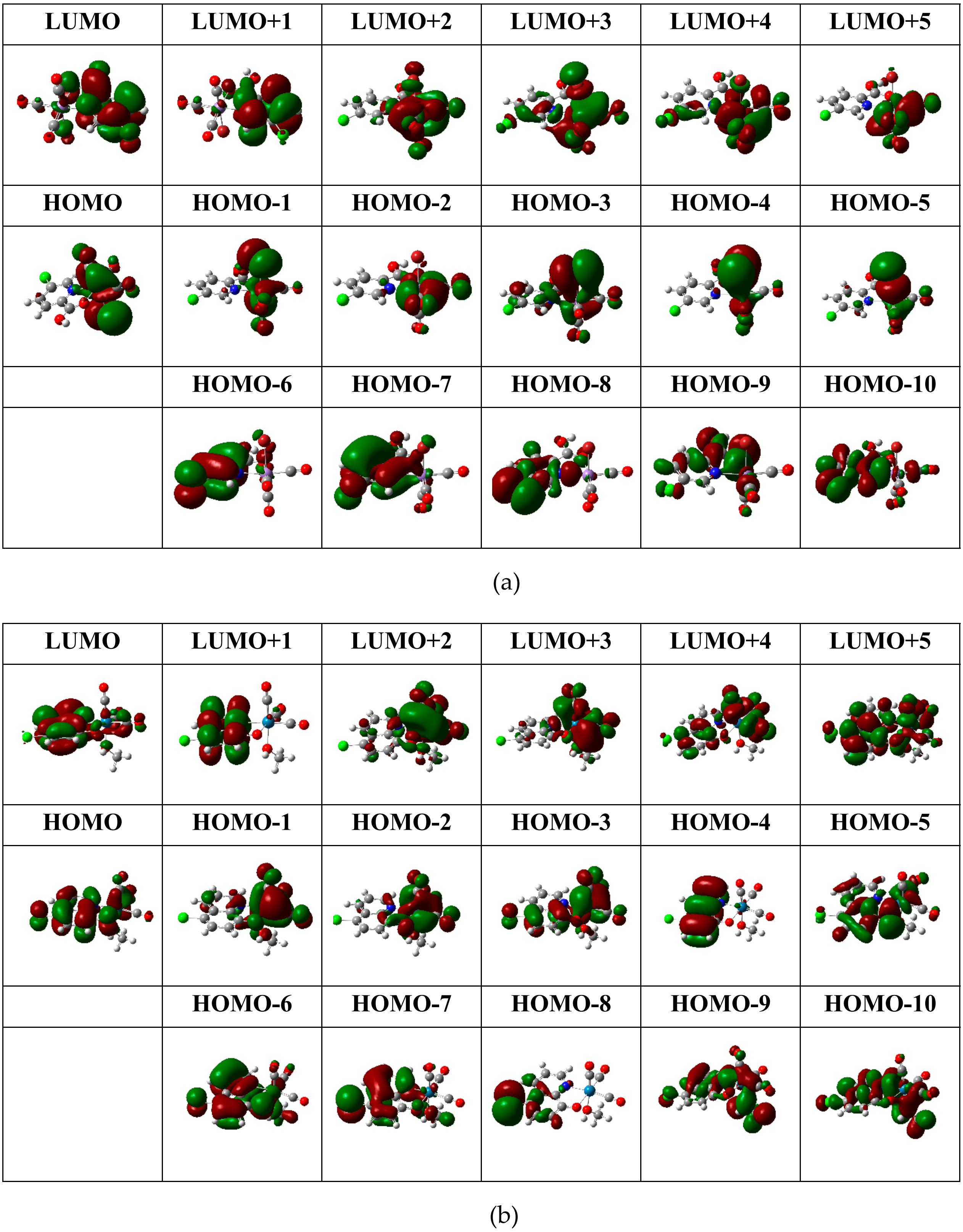

Figure S3. The frontier orbital diagram of the complex: (a) 1 , (b) 2 .

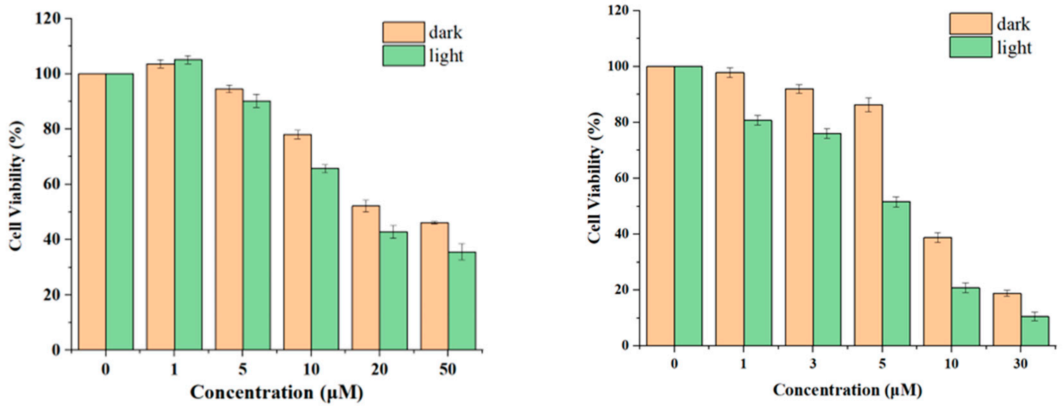

(a) (b)

Figure S4. Cell viability of HeLa cells incubated with complexes 1–2 under both dark and light irradiation conditions: (a) complex 1, and (b) complex 2. Data are averages of three replicates and the errors are standard deviations (n = 3).

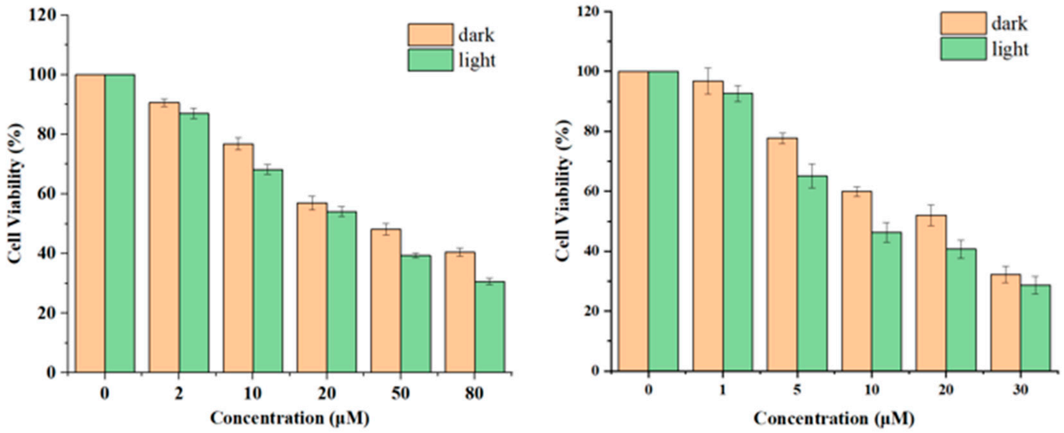

(a) (b)

Figure S5. Cell viability of HL-7702 cells incubated with complexes 1–2 under both dark and light irradiation conditions: (a) complex 1, and (b) complex 2. Data are averages of three replicates and the errors are standard deviations (n = 3).

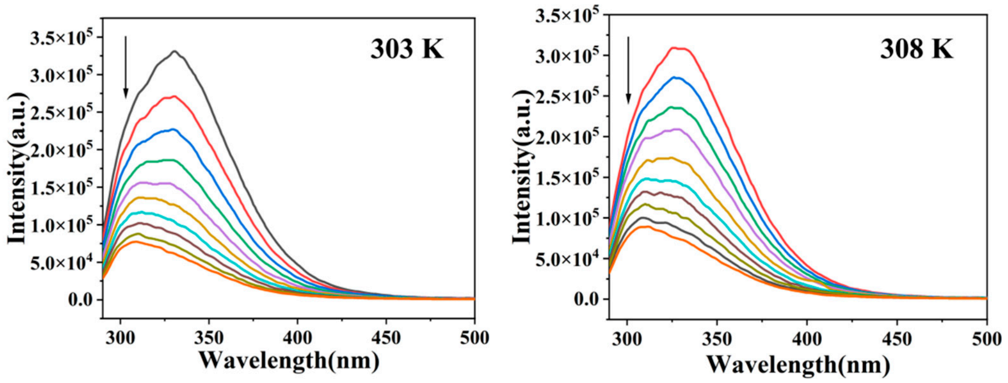

(a) (b)

Figure S6. Fluorescence quenching spectra of HSA in the presence of complex 1 at various concentrations: (a) 303 K, and (b) 308 K.

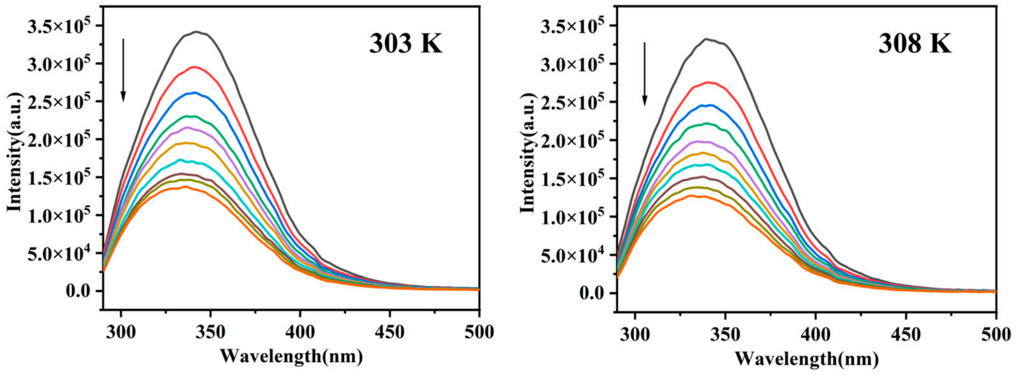

(a) (b)

Figure S7. Fluorescence quenching spectra of HSA in the presence of complex 2 at various concentrations: (a) 303 K, and (b) 308 K.

Table S1. Crystallographic data for complexes 1–2.

**Compound**	**1**	**2**
Empirical formula	$C_9H_4BrClNO_5Mn$	$C_{13}H_9ClNO_5Re$
Formula weight	376.43	480.86
Temperature (K)	296 K	293 K
Wavelength (Å)	0.71076	0.71073
Space group	$C^{-}1$	*I*2/a
a (Å)	19.331(4)	10.0670(3)
b (Å)	12.113(3)	13.7542(4)
c (Å)	13.270(3)	21.5663(6)
α (◦)	90	90
β (◦)	125.432(5)	100.568(3)
γ (◦)	90	90
Volume ($Å^3$)	2532.0(9)	2935.49(15)
Z, Calculated density (Mg/m^3)	8,1.975	8,2.176
Absorption coefficient (mm^{-1})	4.42	8.48
Crystal size (mm)	0.01×0.01×0.01	0.1×0.1×0.1
Theta range for data collection (◦)	2.6-27.2	3.8-28.8
Limiting indices	-21≤h≤24,-15≤k≤15,-17≤l≤15	-14≤h≤13,-19≤k≤19,-30≤l≤30
Goodness-of-fit on F^2	0.98	1.05
Final R indices [I > 2sigma(I)]	R_1= 0.0837, wR_2= 0.0731	R_1=0.0347, wR_2=0.0773
R indices (all data)	R_1= 0.2641, wR_2= 0.1004	R_1=0.0522, wR_2 =0.0883
Largest diff. peak and hole ($e.A^{-3}$)	0.56 and −0.63	2.84 and -1.17

Table S2. Selected bond lengths and bond angles for two complexes from experimental and theoretical calculations.

Parameter	Complex 1			Parameter	Complex 2		
	X-ray data	Singlet state	Triplet state		X-ray data	Singlet state	Triplet state
Mn-N1	2.044(6)	2.107	2.050	Re-N1	2.166(4)	2.214	2.176
Mn-O1	2.069(5)	2.108	1.978	Re-O4	2.213(4)	2.265	2.287
Mn-Br1	2.536(15)	2.598	2.509	Re-O5	2.130(4)	2.145	2.163
Mn-C7	1.783(9)	1.811	1.900	Re-C1	1.893(8)	1.902	1.901
Mn-C8	1.801(9)	1.832	1.940	Re-C2	1.907(6)	1.919	1.916
Mn-C9	1.789(8)	1.816	1.915	Re-C3	1.911(6)	1.929	1.938
O5-C9	1.149(7)	1.148	1.133	O1-C1	1.159(9)	1.154	1.154
∠Mn-C7- O3	178.2(9)	178.8	178.6	∠Re-C1- O1	174.1(6)	178.5	178.4

Table S3. Experimental maximum absorption wavelengths (nm) and major transition bands for two complexes.

Wavelength (nm)				assignment
Complex 1				
exp	cal		f[a]	
265	259	258.81	0.2086	HOMO-6→LUMO+1(40.21%)
				HOMO-2→LUMO+5(10.96%)
				HOMO-1→LUMO+5(21.85%)
				HOMO→LUMO+7(22.86%)
392	340.2	334.62	0.0346	HOMO→LUMO+1(45.51%)

Wavelength (nm)				assignment
Complex 2				
exp	cal		f[a]	
263	246	249.71	0.1440	HOMO-4→LUMO(42.59%)
				HOMO→LUMO+1(15.11%)
347	350	351.97	0.0782	HOMO-1→LUMO(57.53%)
450	455	455.56	0.1004	HOMO→LUMO(69.87%)

Table S4. The orbital energies (in eV) of the highest occupied molecular orbitals (HOMOs), lowest unoccupied molecular orbitals (LUMOs), and other orbitals for the two complexes.

	**Complex 1**	**Complex 2**
LUMO +5	-0.342	-0.158
LUMO +4	-0.842	-0.016
LUMO +3	-1.251	-0.936
LUMO +2	-1.399	-1.029
LUMO +1	-2.022	-1.134
LUMO	-2.982	-2.297
LUMO-HOMO gap	3.456	3.420
HOMO	-6.438	-5.717
HOMO-1	-6.498	-6.561
HOMO-2	-6.883	-6.639
HOMO-3	-7.296	-6.859
HOMO-4	-7.334	-7.562
HOMO-5	-7.890	-8.387
HOMO-6	-8.091	-8.446
HOMO-7	-9.178	-8.863
HOMO-8	-9.347	-8.919
HOMO-9	-9.815	-9.105
HOMO-10	-10.144	-9.412

Table S5. IR spectra of two complexes - theoretical and experimental calculations.

Complex 1		Complex 2		
**exp**	**cal**	**exp**	**cal**	**Assignment**
1650	1622	1580	1609	νPA/Qn
1948	1923	1895	1948	νC≡O
2025	1938		1955	νC≡O
2050	2018	2039	2080	νC≡O

Table S6. K_{sv}, k_q, K_b, n, ΔH, ΔS, and ΔG values for complexes 1–2 with HSA.

	T (K)	$K_{sv}\times10^5$ (M^{-1})	$k_q\times10^{13}$ ($M^{-1}s^{-1}$)	$K_b\times10^6$ (M^{-1})	n	ΔH (kJ mol^{-1})	ΔS (J $mol^{-1}K^{-1}$)	ΔG (kJ mol^{-1})
	298	1.50	1.50	1.61	1.25			-35.31
**1**	303	1.41	1.41	2.35	1.23	69.5	351.7	-37.06
	308	1.13	1.13	4.01	1.34			-38.82
	298	0.79	0.79	0.68	0.87			-33.77
**2**	303	0.58	0.58	0.40	0.90	-170.6	-459.1	-31.47
	308	0.56	0.56	0.07	0.98			-29.18
